# Screening approaches to cardiac amyloidosis in different clinical settings: Current practice and future perspectives

**DOI:** 10.3389/fcvm.2023.1146725

**Published:** 2023-03-09

**Authors:** Angelo Giuseppe Caponetti, Antonella Accietto, Giulia Saturi, Alberto Ponziani, Maurizio Sguazzotti, Paolo Massa, Alessandro Giovannetti, Raffaello Ditaranto, Vanda Parisi, Ornella Leone, Pietro Guaraldi, Pietro Cortelli, Christian Gagliardi, Simone Longhi, Nazzareno Galiè, Elena Biagini

**Affiliations:** ^1^ Cardiology Unit, Cardiac Thoracic and Vascular Department, IRCCS Azienda Ospedaliero-Universitaria di Bologna, Bologna, Italy; ^2^ Department of Experimental, Diagnostic and Specialty Medicine, University of Bologna, Bologna, Italy; ^3^ Department of Pathology, Cardiovascular and Cardiac Transplant Pathology Unit, IRCCS Azienda Ospedaliero-Universitaria di Bologna, Bologna, Italy; ^4^ IRCCS Istituto Delle Scienze Neurologiche di Bologna, Bologna, Italy; ^5^ Department of Biomedical and NeuroMotor Sciences (DiBiNeM), Alma Mater Studiorum-University of Bologna, Bologna, Italy; ^6^ European Reference Network for Rare, Low Prevalence and Complex Diseases of the Heart-ERN GUARD-Heart, Bologna, Italy

**Keywords:** cardiac amyloidosis, heart failure, ATTR, AL, screening, aortic stenosis, nuclear medicine

## Abstract

Cardiac amyloidosis is a serious and progressive infiltrative disease caused by the deposition of amyloid fibrils in the heart. In the last years, a significant increase in the diagnosis rate has been observed owing to a greater awareness of its broad clinical presentation. Cardiac amyloidosis is frequently associated to specific clinical and instrumental features, so called “red flags”, and it appears to occur more commonly in particular clinical settings such as multidistrict orthopedic conditions, aortic valve stenosis, heart failure with preserved or mildly reduced ejection fraction, arrhythmias, plasma cell disorders. Multimodality approach and new developed techniques such PET fluorine tracers or artificial intelligence may contribute to strike up extensive screening programs for an early recognition of the disease.

## 1. Introduction

**Figure 1:**
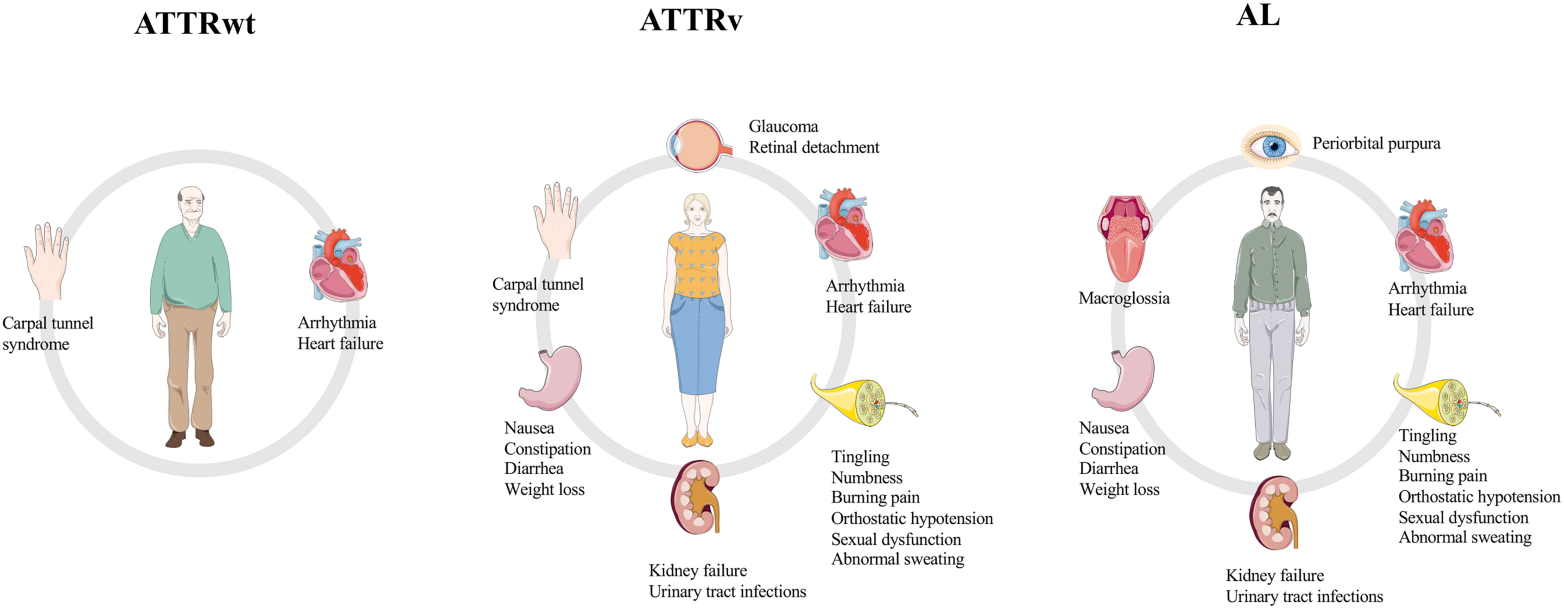
Major manifestations of the three most common subtypes of amyloidosis. ATTRwt, wild-type transthyretin-related amyloidosis; ATTRv, variant transthyretin-related amyloidosis; AL, light-chain amyloidosis. Parts of the figure were drawn by using pictures from Servier Medical Art. Servier Medical Art by Servier is licensed under a Creative Commons Attribution 3.0 Unported License (https://creativecommons.org/licenses/by/3.0/).

Cardiac amyloidosis (CA) is an infiltrative cardiomyopathy characterized by extracellular deposition of an amorphous substance called amyloid, whose formation is secondary to misfolding of different precursors proteins. The most frequent forms of CA are light-chain (AL) and transthyretin-related (ATTR) amyloidosis that can be classified in hereditary (ATTRv) or wild-type (ATTRwt) depending on whether a mutation of TTR gene has been identified (1). Although CA has been historically considered a rare condition, recent advances on medical education and global awareness have conducted to an exponential increase of prevalence of the disease (2). ATTR and AL amyloidosis may share some clinical aspects although organ tropism is generally different (Figure 1). CA may simulate heterogenous cardiac conditions, therefore specific clinical and instrumental features, so called “red flags” (listed in Table 1), have been identified over the years to help physicians to reach a definite diagnosis. The aim of this review was to describe the most common clinical settings in which diagnosis of CA may arise and to examine little-explored fields of interest about CA screening.

**Table 1:** Main red flags related to amyloidosis.

Extracardiac	Cardiac
Polyneuropathy	Low QRS voltage compared to LV thickness
Macroglossia	Pseudo-infarction ECG pattern
Bilateral carpal tunnel syndrome	AV conduction disease
Lumbar spinal stenosis	Granular sparkling
Renal insufficiency, proteinuria	Pericardial effusion
Vitreous deposits	Reduced longitudinal strain with apical sparing
Family history	Increased ECV, elevated T1 values

## 2. Common clinical settings leading to CA diagnosis

### 2.1. CA and orthopedic conditions

It is common knowledge that in patients with ATTR-CA the deposition of amyloid can be observed in soft tissue structures (i.e., ligaments) in addition to myocardial tissue, leading to carpal tunnel syndrome, lumbar spinal stenosis (3, 4), or biceps tendon rupture (5). Among these conditions, carpal tunnel syndrome (CTS) is the most frequent in ATTR-CA population (6). The underlying pathophysiology is still unclear. It has been suggested that repeated mechanical stimuli on ligaments and myocardium could favor amyloid deposition, but there is no such scientific evidence. An Italian study found that CTS history was present in 14% of ATTRv and 25% of ATTRwt patients, significantly higher compared to general population (4.1%). CTS was often bilateral, and it usually occured 5 to 9 years prior to CA diagnosis. In AL patients, no signs of increased incidence of CTS were observed (7). In the last years, researchers have been developing many strategies for earlier disease detection, including carpal tunnel release (CTR) specimens' analysis. For instance, in a cohort of 98 adults undergoing CTR, 10 patients were found to have a positive tenosynovial biopsy for amyloid presence (7 ATTR, 2 AL and 1 untyped) after Congo red staining. Only two patients had cardiac involvement (1 AL amyloidosis and 1 ATTR) at the time of screening for CA, for an overall yield of 2%. To note, only those with a positive tenosynovial biopsy underwent a screening for CA, and this could explain the low prevalence of cardiac involvement in this population (8). In 2019, Fosbøl et al. attempted to esteem the risk of future amyloidosis associated with CTS using a Danish nationwide registry of patients who underwent CTR surgery and comparing them to a sex- and age-matched cohort from the general population. They found that the absolute incidence of future diagnosed amyloidosis among CTS patients is low, but significantly higher compared to general population (cumulative incidence of 0.10% by 10 years in the CTS group vs. 0.006% among control subjects). Also, CTS population showed a higher risk of heart failure (HF), with a hazard of 1.5 times that of control subjects and a cumulative incidence of 4% at 10 years. However, in this study the association of CTS with heart failure did not negatively affect the early mortality when compared to a HF population without CTS (9). These results have been recently confirmed in a Danish study (10) which evaluated the association of previous CTS surgery and cardiovascular outcomes in patients who underwent permanent pacemaker implantation. In fact, authors found that previous CTS surgery was associated with increased risk of new-onset HF and increased risk of diagnosed amyloidosis after pacemaker implantation. Also, no association between previous CTS surgery and increased mortality could be established. Lately, Westin et al. published the results of the CACTUS (Cardiac Amyloidosis Carpal TUnnel Syndrome) study, a trial designed to determine the prevalence of undiagnosed CA in patients 5–15 years after bilateral CTR (11). They identified 250 subjects (50% females) with a median age of 70 years old with prior bilateral CTR and evaluated them for CA by performing echocardiography, bone scintigraphy, and monoclonal protein studies. CA was diagnosed in nearly 5% of patients. All cases were wild-type transthyretin amyloidosis (ATTRwt). As underlined by the authors, when focusing on men ≥70 years, prevalence of undiagnosed CA rises to 21.5%, therefore playing a potential role for systematic screening in this population. Notably, most of ATTRwt diagnosis (10 of 12 patients) had very early-stage disease. Globally, orthopedic conditions are very common in patients affected by CA (predominantly ATTR). Indeed, Rubin and colleagues have found that 25.9% and 18.8% of patients with ATTRwt-CA and ATTRv-CA, respectively, underwent hip or knee arthroplasty. Compared to the general population, surgical procedures were significantly more common among patients with ATTR-CA (hip arthroplasty: RR: 5.61, 95% CI: 2.25–4.64; knee arthroplasty: RR: 3.32, 95% CI: 2.25–4.64). As for other musculoskeletal disorders, arthroplasty occurred significantly before ATTR-CA diagnosis, and it was not significantly more common in AL patients compared to general population (12). Consequently, a history of a multidistrict orthopedic surgery should be definitely considered a red flag for suspicion of ATTR amyloidosis.

### 2.2. CA and aortic stenosis

It is well known that aortic stenosis (AS) and ATTRwt share the same epidemiological background as their prevalence is particularly high in male octogenarians, but whether this represents a simple epidemiological association, or a causal relationship is still an unanswered question. As previously summarized by Rapezzi et al. (13), there are three main hypotheses that may explain the association between those two conditions: (1) an age-dependent incidence of both diseases, thus involving the same subgroups of the population; (2) amyloid deposition involves the aortic valve, promoting or accelerating valvular degeneration; (3) AS itself can possibly trigger or cause transthyretin cardiac amyloidosis by increasing left ventricular (LV) wall shear stress. The question is relevant as both diseases carry a high burden of morbidity and mortality and the effect of their coexistence on patients' prognosis has not fully clarified, although recent data suggest that treatment options of AS improve survival even in patients affected by CA and consequently they should not be withheld (14). Moreover, along with population ageing, the absolute number of people affected is considerable and represents a public health issue in most developed countries.

ATTRwt's prevalence among aortic stenosis cohorts is variable between 4% and 16%, mainly influenced by the mean age of the studies' population and by the diagnostic criteria or imaging exams used for diagnosis (15). In a very early study, Treibel and colleagues (16) prospectively enrolled 146 patients with severe AS requiring surgical valve replacement and performed intraoperative myocardial biopsies, finding a prevalence of 6% of occult transthyretin CA. In another small single-centre prospective study, Longhi et al. (17) performed a 99mTc- 3,3-diphosphono-1,2-propanodicarboxylic acid (DPD) scintigraphy to detect occult ATTR-CA based on echocardiographic features. They applied an “echocardiographic red flags” approach to 43 elderly patients with AS referred for aortic valve replacement (surgical or transcatheter), including one or more of these features: increased thickness of atrioventricular valves, interatrial septum or right ventricular free wall, pericardial effusion, and myocardial granular sparkling. 5 of the 43 patients enrolled had at least one red flag at echocardiogram and all of them showed high cardiac uptake at 99mTc-DPD scintigraphy (prevalence = 11%). Similar percentages are reported in more recent clinical studies in which the systematic search of CA associated with AS was made by using multimodal imaging techniques (14, 18). To note, they all confirmed that bone scintigraphy shows a better sensitivity to detect ATTR-CA than cardiac magnetic resonance (CMR), even in presence of valvular abnormalities. Recently, computed tomography (CT) scan has been addressed as an additional emerging tool for identifying CA in patients with AS. In a single tertiary referral center study, an additional post-contrast acquisition for extracellular volume (ECV) evaluation was added to routine CT scan evaluation for transcatheter aortic valve replacement planning. All patients underwent bone scintigraphy as well. CT-measured ECV showed a good correlation with Perugini score, suggesting that this technique can reliably detect AS-CA and quantify the degree of infiltration (19).

Recently, researchers' efforts have been focused on identifying clinical, biochemical, electrocardiographic, and echocardiographic features that could help clinicians to raise the suspicion of underlying CA in patients affected by AS. Although some instrumental characteristics can be shared by both conditions (particularly LV hypertrophy), the identification of multiple distinctive features (listed in Table 2) should promptly trigger a diagnostic work-up for CA. A scoring system called RAISE was developed in a multicenter international study enrolling patients referred for transcatheter aortic valve replacement (14), including five domains: LV remodeling (LV hypertrophy and/or diastolic dysfunction, 1 point), older age (>85 years old, 1 point), cardiac injury (serum high sensitivity troponin I >20 ng/L, 1 point), systemic involvement (carpel tunnel syndrome, 3 points), and electrical remodeling (right bundle branch block, 2 points or low QRS voltage, 1 point). Authors suggest that scores of ≥2 points would promote further investigation by bone scintigraphy and light-chain assessment thanks to a high sensitivity (93.6%), but scores ≥3 points shows a better specificity (84% vs. 52%).

**Table 2:** Characteristic parameters of cardiac amyloidosis in aortic stenosis.

Clinical	Male sex
	Age >80 years
	Carpal tunnel syndrome (or multidistrict orthopedic surgery)
ECG	Atrial fibrillation
	Atrio-ventricular and intra-ventricular conduction abnormalities
	Discrepancy between left ventricular mass and voltages
	Q waves (pseudo-infarction pattern)
Echocardiography	Severe left ventricular hypertrophy
	Low flow-low gradient aortic stenosis
	Right ventricle hypertrophy (>5 mm)
	Marked diastolic dysfunction (*E*/*e*′ > 15)
	Severe atrial enlargement
	Very reduced S′ wave at mitral annulus Tissue Doppler
	Reduced global longitudinal strain with “apical sparing” pattern

### 2.3. CA and bone scintigraphy for non-cardiac reasons

Studies investigating prevalence of cardiac uptake in individuals undergoing bone scintigraphy for oncologic or rheumatologic reasons provide the most reliable information about the prevalence of CA in the general population. Recently, Aimo et al. performed a systematic meta-analysis of published studies (*n* = 5) and they found a prevalence of CA among patients undergoing bone scintigraphy for non-cardiac reasons of 1%, with a higher likelihood in men and increasing prevalence with age (20). The largest study in this setting was published in 2014 by Longhi and colleagues (21). Authors retrospectively analyzed 12,400 99mTc-DPD bone scans made for oncologic (95%) or rheumatologic (5%) indications in people aged >65 years: unexpected myocardial tracer uptake was present in 0.36% (*n* = 45) of patients, reaching 1.4% among male octogenarians. Of them, 14 patients with a median age of 82 years underwent a comprehensive cardiological evaluation, including ECG, echocardiogram and endomyocardial biopsy in selected cases. Five patients eventually received a diagnosis of ATTRwt, and 1 patient was found to have ATTRv. Despite the small number of definite diagnoses of ATTR-CA, this study highlighted for the first time the possibility of pre-clinical identification of patients affected by ATTR-CA in an unselected population. A subsequent study investigated the prevalence of myocardial tracer uptake in bone scans in an older cohort, finding a higher percentage of unexpected positive scans (3.88% of males over 75 years old) (22). Interestingly, cardiac uptake was associated with a higher risk of HF hospitalization during follow-up (OR: 2.60, 95% CI: 1.09–5.74, *p* = 0.022).

A recent Australian study found a prevalence of 0.43% of cardiac uptake in a heterogeneous cohort of over 3,000 patients who underwent 99mTc-hydroxy-methyl-diphosphonate (HMDP) bone scans for non-cardiologic reasons (23). Data confirmed that prevalence increases with age, and it is higher in males than females (1.44% vs. 0.17% in subjects >65 years old). Notably, nearly 29% of patients (998/3472) were <65 years old but none of them had cardiac uptake, corroborating the idea that positive bone scans in younger individuals are quite rare.

In this study, a positive correlation between positive scans (visual score ≥2) and septal wall thickness measured by echocardiography was observed, as well as between LV mass and the amount of HMDP uptake (measured by heart to whole body ratio); conversely, the degree of tracer uptake correlated negatively with LVEF, suggesting that higher HMDP uptake is associated with more advanced disease. However, in this population positive HMDP scans were surrogate marker of ATTR-CA, as AL amyloidosis was not systematically excluded, representing a possible confounding variable.

### 2.4. CA and heart failure

Nowadays, heart failure (HF) represents a considerable public health issue as it affects approximately 5% of the general population aged ≥60 years and HF with preserved ejection fraction (HFpEF) accounts for near the half of HF diagnosis in US (24). To date, few treatments have demonstrated to reduce cardiovascular events or mortality in HFpEF overall population. For such reason, it is crucial to exclude a possible specific cause of HFpEF, especially if disease-modifying therapies are available.

HFpEF strongly correlates with CA, since its pathophysiology is characterized by increase of myocardial stiffness causing an impaired LV relaxation, leading to high LV end-diastolic pressure and impaired ventricular filling (25). Since Mohammed et al. (26) have found that the age- and sex-adjusted prevalence of wild-type TTR amyloid was higher in HFpEF patients than in control subjects (odds ratio: 3.8, 95% confidence interval: 1.5 to 11.3; *p* 0.03) on histological screening in LV autopsy specimens, CA has been found to be a common etiology in such patients within several subsequent clinical studies. It should be noted that small quantities of transthyretin amyloid are commonly seen in the autoptic analysis of hearts of elderly patients, but their contribution to the burden of HFpEF is unclear. Indeed, whether the neurohumoral dysregulation and metabolic imbalance seen in HFpEF population can cause a destabilization of wild-type transthyretin molecules or on the other side the cytotoxic and profibrotic effect of the primary deposition of amyloid fibrils can cause the diastolic dysfunction underlying the disease is still an open question. In 2015, Gonzalez-Lopez and colleagues (27) prospectively screened a population of 120 patients aged >60 years old admitted to the hospital for HFpEF by performing 99mTc-DPD scintigraphy. They found an intense cardiac uptake (grade 2 or 3) in approximately 13% of cases; of these, no one was found to carry a genetic mutation of transthyretin gene. Notably, only 4 patients underwent to endomyocardial biopsy confirmation and light-chain amyloidosis (AL) was not systematically excluded. Afterwards, a multimodal cardiovascular imaging approach was tested to phenotype a population of patients over 65 years old with HFpEF in 2016 (28). In this study, 49 patients admitted to the hospital for signs and symptoms of HF and a LV ejection fraction >45% with no evidence of coronary artery disease underwent a complete evaluation with all clinical available cardiovascular imaging modalities, including echocardiography, cardiac magnetic resonance and 99mTc-DPD scintigraphy. The authors found a prevalence of 18% in ATTR-CA, similarly to Gonzalez-Lopez's group findings, 12% of AL cardiomyopathy and 6% of hypertrophic cardiomyopathy. In this paper, a combination of CMR and 99mTc-DPD scintigraphy, in addition to biochemistry, clinical and echocardiographic evaluation, appeared to expand clinicians' possibility to characterize this population, contributing to the current idea that HFpEF is a complex and heterogeneous group in etiology and pathophysiology.

More recently, a Swedish study investigated the prevalence of ATTR-CA in a heart failure population with myocardial hypertrophy (29). Indeed, the investigators performed a 99mTc-scintigraphy in an unselected cohort of elderly patients (median age 84 years) with heart failure, irrespective of ejection fraction, and increased wall thickness (defined as interventricular septum >14 mm). Fourteen of the 86 (20%) investigated patients had a cardiac uptake of grade 2 or 3 at bone scintigraphy, while 5 patients had an uptake of grade 1. Only one patient was found to carry a mutation in TTR-gene. All patients were evaluated with blood and urine samples to exclude AL amyloidosis. Although a different patient recruitment protocol, these data appear to be in line with the previous ones. In fact, this study has remarkably focused on patients with myocardial hypertrophy, suggesting that the disease could not be confined only to HFpEF, as more than half of the cohort had an ejection fraction <50%.

### 2.5. CA and plasma cell disorders

The incidence of AL amyloidosis is ≈0.8%/100.000 population and symptomatic cardiac involvement seems to be present in 30% to 50% of cases (30). Most patients do not present multiple myeloma and in most cases the underlying plasma cell dyscrasia would be classified as monoclonal gammopathy of undetermined significance. AL amyloidosis may indeed complicate approximately 10%–15% of cases of plasma cell disorders (31). Cardiac amyloidosis is one the major parameter to impact on survival in AL amyloidosis (32), therefore an early recognition of signs of cardiac disease must represent an essential goal in order to start as soon as possible a specific therapy.

Nowadays, screening a plasma cell dyscrasia by assessing serum and urine protein electrophoresis with immunofixation and serum free light chains represents an imperative part of the diagnostic work-up in patients with suspected CA (33). Conversely, a systematic consensus on how and when to screen CA in plasma cell disorders is lacking. Patients with AL amyloidosis without cardiac involvement at baseline should be followed at least once a year in order to rule out the onset of CA, especially in those patients without a good hematologic response to treatment (34). Also, patients with a monoclonal gammopathy without AL amyloidosis should undergo to periodical cardiologic assessments, although the epidemiologic burden of the disease makes it challenging. Therefore, some authors suggest to screen pre-symptomatic amyloid organ involvement simply by assessing laboratory biomarkers (e.g., NT-proBNP, albuminuria, alkalin phosphatase) (35). Cardiac biomarkers (NT-proBNP and troponin) play an essential role on the diagnosis and the estimation of prognosis in AL-CA (32, 36), even in the absence of overt cardiac involvement defined by standard criteria. Recently, Sherpley et al. conducted a prospective observational study on Mayo stage I patients (N-terminal pro b-type natriuretic peptide <332 ng/L, high sensitivity cardiac troponin <55 ng/L) without cardiac involvement on echocardiogram. All patients also underwent to CMR, which documented signs of CA in 28% of patients. On multivariate analysis, N-terminal pro b-type natriuretic peptide >152 ng/L and cardiac involvement on CMR were prognostic. Interestingly, not all patients with elevated NT-proBNP had abnormal CMR and vice versa. Authors suggest that NT-proBNP may be detecting cardiac damage by light chain proteotoxicity before structural amyloid deposition (potentially detected by CMR), while some patients may have non-proteotoxic light chains where the structural changes are already evident on CMR. In conclusion, this study underlines the importance of combining standard cardiologic evaluation to NT-proBNP and CMR, which both play a complementary and crucial role on defining cardiac involvement (37).

### 2.6. CA and LV hypertrophy

LV hypertrophy is certainly one of the most recognized features of CA, although its presence is shared by many other conditions such as hypertrophic cardiomyopathy, Anderson-Fabry disease and hypertensive heart disease. Many strategies have been developed to detect CA in this context. For instance, an Italian study prospectively enrolled 343 patients aged ≥40 years referred to a Tertiary Centre with a previous echocardiographic diagnosis of hypertrophic cardiomyopathy (38). All patients underwent to a next-generation sequence genetic testing, of which 11 (3.2%) resulted positive for a TTR gene mutation and 1 for ApoAI mutation. Among remaining mutation-negative patients, authors investigated further with laboratory analyses, bone scintigraphy, and fat abdominal biopsy those who presented at least one “red flag” for CA, including pericardial effusion, symmetric LV hypertrophy or granular sparkling texture of the myocardium, thus increasing the number of total diagnoses of CA to 32 patients (9% of study population). Remarkably, prevalence of CA increased with age, ranging from 1% at ages 40–49 years to 26% above 80 years. The role of echocardiographic “red flags” has been widely investigated as a preferable approach for the diagnosis of CA. In 2020 Boldrini et al. (39) have proposed a multiparametric echocardiographic score with a very good diagnostic accuracy (area under the curve 0.87, 95% CI: 0.85–0.90) including relative wall thickness, *E*/*e*′, TAPSE, longitudinal strain and “apical sparing” (defined as relative apical longitudinal strain >1.0, using the equation average apical LS/average basal LS + mid-LS) (40), within a sub-cohort of patients with LV hypertrophy and suspicion of CA. For the record, apical sparing More recently, the investigators of the AC-TIVE Study (41) aimed to assess the prevalence of CA in a much more unselected population. In fact, in the Phase 1 they evaluated more than 5,000 consecutive patients aged ≥55 years undergoing routine echocardiography to investigate the usefulness of echocardiographic “red flags” in detecting AC among general population by looking for hypertrophic and non-dilated left ventricles with preserved ejection fraction in the first place (“CA compatibles”). Of the initial study population, 1,169 (22%) exams were CA-compatibles, and among these, 381 exams (33% of CA compatibles—7% of the total) were “CA suggestive”, based on the presence of well-known clinical or echocardiographic “red flags”. Notably, in this context thickening of the interatrial septum was the most frequent echocardiographic feature, followed by pericardial effusion, restrictive LV filling pattern, granular sparkling appearance of the myocardium, thickened atrio-ventricular valves and apical sparing pattern upon speckle-tracking analysis. In Phase 2 of the AC-TIVE study (4) they investigated the prevalence of CA among patients with echocardiographic red flags (the aforementioned “CA suggestive” population) by following the conventional diagnostic flow-chart for CA. Among patients who completed the diagnostic work-up (*n* = 217), 62 received a final diagnosis of CA with an estimated prevalence of nearly 29%. In this phase, statistical analyses showed that apical sparing alone or a combination of at least two red flags, had a diagnostic accuracy >70%, providing a guide for physicians in proceeding with further tests for AC when performing echocardiography for any reason. CMR may play a supplementary role in the instrumental evaluation of LV hypertrophy. In fact, in addition to the morphological and functional assessment of myocardial walls, CMR offers tissue characterization by measuring native T1 and native T2 (possibly with corresponding quantitative values in T1 mapping and T2 mapping) and extra-cellular volume (ECV). Those three measures have been proven in many studies to add both a diagnostic and prognostic value, even in distinguishing AL and ATTR amyloidosis (42, 43).

**Figure 2:**
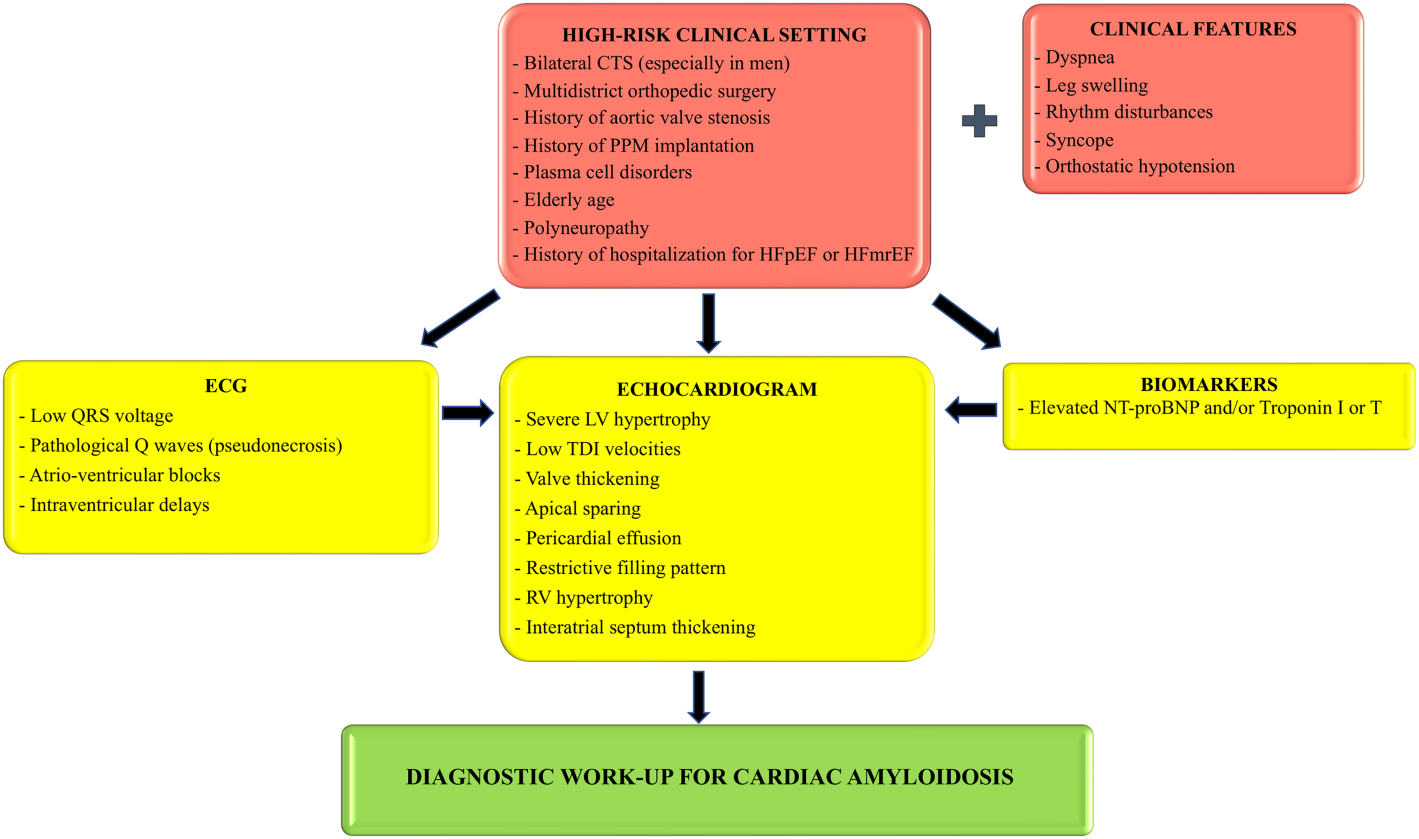
Proposed screening algorithm for patients belonging to high-risk clinical settings. ECG, echocardiogram and cardiac biomarkers can all be used as a first step methodic in the suspicion of cardiac amyloidosis, although echocardiogram is imperative before starting a diagnostic work-up. CTS, carpal tunnel syndrome; PPM, permanent pacemaker; HFpEF, heart failure with preserved ejection fraction; HFmrEF, heart failure with mildly reduced ejection fraction; LV, left ventricular; TDI, tissue Doppler imaging; RV, right ventricular.

Figure 2 summarizes our proposed algorithm for the screening of CA in different clinical scenarios.

## 3. Future perspectives

**Figure 3:**
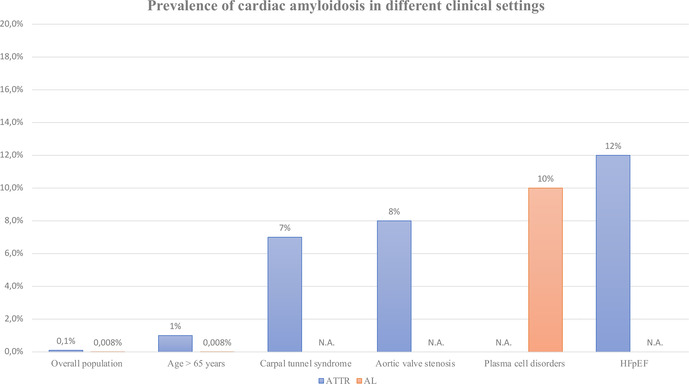
Prevalence of cardiac amyloidosis in different clinical settings. Data represent a rough estimation according to the main published evidence in literature. Not applicable (N.A.) was used in case of missing solid data. ATTR, transthyretin-related amyloidosis; AL, light-chain amyloidosis; HFpEF, heart failure with preserved ejection fraction.

As previously explained, the epidemiology of CA is vertiginously evolving, as if a “Pandora's box” was just opened by international scientific community. Figure 3 recapitulates the prevalence of this condition fitting to the different clinical settings we investigated in this review. Yet, various clinical and experimental fields may still be explored to better define the most appropriate approaches to screen and then manage the disease.

For instance, atrial and ventricular arrhythmias and conduction disturbances are frequently found in patients affected by CA, nonetheless a systematic screening in this setting—still lacking—could offer further insights on the matter. Above all, atrial fibrillation and atrio-ventricular delays appear to be the most common (44, 45). Nevertheless, non-sustained ventricular arrhythmias are rather frequent and carry a debatable prognostic role (46), probably not higher as usually thought, as showed in a multicenter retrospective Italian cohort of 181 patients affected by CA (51 AL and 130 ATTR), in which the presence of non-sustained ventricular arrhythmias was related to the severity of disease but not with mortality in the total population and in each amyloidosis subtype (47). The underlying pathophysiology is not completely understood as there is no convincing histological evidence of amyloid involvement of the conduction system tissue (48). Most likely, the pathogenesis of arrhythmias in CA is multifactorial (49). Amyloid deposition has been shown to cause wall thickening and disarray of myocardial fibers, which *per se* can disrupt the transmission of electrical impulses along conduction fibers. Also, there is an emerging hypothesis for a cytotoxic role of transthyretin molecule involving the dysregulation of intracellular calcium signaling causing action potential prolongation, in addition to oxidative stress and apoptosis (50). Furthermore, amyloid deposition from transthyretin monomers is thought to be neurotoxic in ATTRv patients and can cause the drive loss of sympathetic nerve fibers, which may contribute to arrhythmogenesis (51). Interestingly, electrophysiologic studies have shown not only prolonged AH intervals in CA patients, but also abnormal HV intervals, even in presence of a relatively normal QRS duration, reflecting an extended conduction system disease which determines an equal delay in both ventricles. This suggests that a narrow QRS does not exclude an intranodal conduction disease in this subset of patients and therefore should be considered in the diagnostic work-up of syncope (52). As result, permanent pacemaker implantation is often required. About 13% of patients affected by ATTRwt have a history of pacemaker implantation prior to diagnosis (53). In a recent prospective study conducted by Porcari et al. on a cohort of patients affected by ATTR-CA or AL-CA, 8.9% of patients underwent to pacemaker implantation during a median follow-up of 33 months, with an overall median time to PPM implantation at 18 months. An history of atrial fibrillation, a longer PR interval and QRS >120 ms on baseline ECG appeared to be independent risk factors for pacemaker implantation (54). In accordance with those data, a systematic search for CA (especially in presence of other “red flags”) among patients with implanted devices could represent an intriguing field of research.

Among the instrumental methods to screen and diagnose CA, positron emission tomography (PET) has been gaining share, although its use on clinical practice is still hypothetical. In a screening setting, PET tracers may have a superior role both on detection of amyloid deposits and differentiation between ATTR and AL. For instance, 18F-Fluorbetaben was first tested by Law et al. in a small group of patients and showed higher standardized uptake values (SUV) in cardiac amyloidosis (both AL and ATTR forms) than hypertensive heart disease (55). A recent study by Genovesi et al. reported that 18F-Fluorbetaben kinetic of myocardial retention in AL-CA was much longer than ATTR-CA and in control subjects (56). 18F-Flutemetamol has only been tested in a small study which reported myocardial uptake exclusively in amyloid cardiomyopathy and not in the control group. Moreover, TBR (target-to-background ratio, the ratio between myocardial SUV and blood-pool SUV) was particularly high in the single patient with AL-CA enrolled in the study (57). An ongoing multicenter phase 2 study is evaluating the repeatability of organ-specific quantitation of 124I AT-01 tracer using PET/CT in ATTR and AL amyloidosis (NCT05235269). This radiotracer represents a novel pan-amyloid binding imaging agent. Preliminary data suggest that it may be useful to identify pre-symptomatic stage of CA (for instance in ATTRv carriers) and to detect amyloid deposits within the whole body, potentially allowing to replace tissue biopsy for the assessment of organ involvement, especially in AL amyloidosis.

Lastly, artificial intelligence (AI) represents a widespread technique whose application in cardiology has been promisingly tested and approved in several contexts (58). AI describes a computational program that can perform tasks that are normally characteristic of human intelligence. In medicine, this typically involves data, health records or information extracted from images, used to predict a likely diagnosis, identify a new disease, or select a best choice of treatment. Grogan et al. collected 12-lead ECG data from 2,541 patients with AL-CA or ATTR-CA referred to Mayo Clinic between 2000 and 2019, matched for age and sex with 2,454 controls. A subgroup of 2,997 cases and controls were used to train a deep neural network to predict the presence of CA. 426 (84%) of the patients with CA were detected by the model, predicting the presence of CA more than 6 months before the clinical diagnosis in 59% of cases (59). A first pragmatic trial will be soon conducted at Mayo Clinic (NCT05557162) to prospectively evaluate the use of the AI-ECG dashboard in everyday practice. The principle aim of the study will be to prove if an alert system AI-ECG and enhanced algorithms enable earlier diagnosis of CA on compared to standard practice arm. Further clinical trials need to be performed, hopefully by combining multi-modality imaging, with the ultimate goal of making diagnosis of CA ever more extensive and accessible.

## 4. Conclusions

CA is a threatening disease which may be hiding in multiple conditions such as carpal tunnel syndrome, aortic stenosis, heart failure, plasma cell disorders. In those scenarios, detecting the disease may be difficult because some features overlap each other, therefore the application of a multimodality imaging approach along with the awareness and recognition of specific characteristics, so-called “red flags”, are fundamental. Although the cost-effectiveness of a screening strategy in CA has not been scientifically proven yet, this extensive and meticulous approach can be beneficial for various potential reasons: - intercepting a pre-symptomatic phase in which “disease-modifying” therapies may act the most; - recognizing an underlying neoplastic process such as AL amyloidosis; - identifying hereditary CA from probands to siblings; - better understanding (and maybe predicting) the cardiac disturbances of recruited patients; - tailoring supportive cardiac and non-cardiac therapy within the peculiar CA pathophysiology and natural history. On this basis, the efforts of scientific community must continue along the direction of increasing knowledge of the disease.
